# Efficient Hydrolysis of Fish Parvalbumin by Marine Bacterial Protease VSP2V‐280: Allergen Removal

**DOI:** 10.1002/fsn3.4729

**Published:** 2025-01-07

**Authors:** Junlan Zhou, Yue Bai, Yuan Gao, Huili Tian, Ming'ao Wang, Xinxin Kang, Lei Zhang, Mingsheng Lv, Shujun Wang

**Affiliations:** ^1^ Jiangsu Key Laboratory of Marine Bioresources and Environment/Jiangsu Key Laboratory of Marine Biotechnology Jiangsu Ocean University Lianyungang China; ^2^ Co‐Innovation Center of Jiangsu Marine bio‐Industry Technology Jiangsu Ocean University Lianyungang China

**Keywords:** allergen removal, cloning and expression, enzymatic properties, parvalbumin, protease

## Abstract

Parvalbumin is a major allergen in fish. However, there is currently no effective and safe way to remove this allergen from fish. In this study, protease gene VSP2V‐280 of marine bacteria *Virgibacillus* sp. SP2 was cloned and expressed. The protease enzyme showed maximum activity at 50°C and pH 10.0. Ca^2+^ and Cu^2+^ promoted the enzyme. The enzyme showed good parvalbumin degradation efficiency in fish. Based on the gel analysis, when 0.3 mg/mL of parvalbumin was incubated with protease VSP2V‐280 (30 U/mL) containing 1 mM Ca^2+^ for 3 h, the parvalbumin removal rate reached 97%. The enzyme was further used for parvalbumin removal from *Ctenopharyngodon idella* , *Pelteobagrus fulvidraco* , *Parabramis pekinensis* , and *Carassius auratus* . The parvalbumin removal rate reached 93% in 4 h at an enzyme dosage of 72 U/mL. The study showed the potential of VSP2V‐280 to remove parvalbumin from aquatic products.

## Graphical abstract

*Virgibacillus* sp. SP2 produces protease that specifically removes parvalbumin. Protease gene VSP2V‐280 of *Virgibacillus* sp. SP2 was cloned and expressed in *Escherichia. coli* and screened for removal of parvalbumin. Ca^2+^ had a significant activating effect on VSP2V‐280 and it effectively promoted the removal of parvalbumin. VSP2V‐280 effectively removed parvalbumin from freshwater fish.

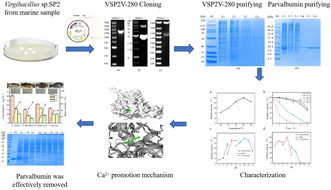

## 1 Introduction

Food allergy refers to the specific immune response caused by the ingestion of certain food components, and it has become a common public health concern throughout the country (Foong et al. 2021; Locke et al. 2023; Wai et al. 2019). According to epidemiological surveys, nearly 5% of adults and 8% of children worldwide are affected by adverse reactions of immunogens originating from foods (Arasi et al. 2023; Tang et al. 2019; Venter, Smith, and Fleischer 2023).

Aquatic products are rich in protein and are important food materials for human beings (Beale, Jeebhay, and Lopata 2009; Khora 2016). However, the allergens in the aquatic products can cause severe allergic reactions in susceptible people. Therefore, fish and crustacean products have been declared as allergic foods by the Food and Agriculture Organization of the United Nations and the World Health Organization (Kim et al. 2008; Ruethers et al. 2018). So far, up to 11 kinds of allergens have been identified in fish, such as parvalbumin, β‐enolome, aldolase, and tropomyosin. Among these allergens, parvalbumin is found in 97% of fish (Permyakov, Uversky, and Permyakov 2017; Sicherer et al. 2020) and 95% of fish allergies are caused by parvalbumin (Mukherjee et al. 2023, 2024). Parvalbumin has many subtypes and it belongs to calcium‐binding protein family, which is one of the largest protein families of food allergens calcium‐binding proteins (Aksun 2022; Jiang et al. 2024; Veshchitskii and Merkulyeva 2023). These proteins usually exist in skeletal muscle cells. Parvalbumin was first discovered by Aas et al. from cod and named as Gad c 1 (Aas and Elsayed 1969). Parvalbumin usually has a molecular weight of 10–13 kDa and an isoelectric point of 4.1–5.2. Furthermore, it is easily soluble in water, resistant to high temperature and not susceptible to denaturation (Mahdi and Ribi 2019; Pekar, Ret, and Untersmayr 2018).

In recent years, exploring the ways to reduce the sensitization of aquatic products during processing has become a research hotspot (Finegold 2022; Tsabouri et al. 2012). At present, the main methods to reduce food allergens in aquatic products include heat treatment, irradiation, ultra‐high pressure, and enzymatic treatment (Tsabouri et al. 2012). Heat treatment is a common food processing method, which destroys the tertiary structure of allergen protein and reduce its ability to bind to human IgE, thus reducing its sensitization (Bhat et al. 2021). Kubota et al. studied the sensitization of parvalbumin in Pacific mackerel and reported decreased reactivity of IgE for parvalbumin after heat treatment of fish tissue at 140°C (Kubota et al. 2016). Fang et al. boiled and baked (180°C) tropomyosin, a major oyster allergen, and found higher IgE reactivity as compared to untreated tropomyosin (Fang et al. 2018). This suggests that heating may lead to formation and degeneration of protein polymers. Lu et al. compared the effects of microwave, ultrasound, and high temperature and pressure processing methods on digestibility and sensitization of tropomyosin in Mitten crab (Yufeng et al. 2023). The results revealed that high temperature and pressure was the most effective way to accelerate tropomyosin digestion and reduce the binding ability of IgE through digestive enzymes. Li et al. used electron beam irradiation for carbonylation of fish parvalbumin, thereby reducing the sensitization of turbot fish. However, long heating time and high radiation dose have been reported to reduce the allergen sensitization as well as the quality of food to some extent (Li et al. 2014). Kobayashi et al. developed a simple and reliable sodium dodecyl sulfate (SDS)‐PAGE method to quantify parvalbumin in fish and reported that the parvalbumin content varied significantly depending on the fish and the location of muscular cells (Kobayashi et al. 2016). In recent years, enzymatic removal of allergens from food has attracted great attention due to several advantages of this process, such as strong specificity, high removal rate, mild processing conditions, and less investment required for equipment. Enzymatic hydrolysis is the process of hydrolyzing macromolecular allergens into small fragments or peptides, destroying their spatial structure and epitopes to reduce their sensitization (Khan et al. 2019). Yang et al. expressed the alkaline protease BaApr1 obtained from *Bacillus altitudinis* W3, which showed a good desensitization effect on milk protein (Yang et al. 2020). This indicated the potential application value of AprEs for desensitization of milk protein.

Marine microorganisms are one of the important sources of alkaline protease (Ameen, AlNadhari, and Al‐Homaidan 2021). Compared to the proteases of terrestrial microorganisms, the proteases produced by marine microorganisms show higher salt tolerance, wider range of temperature tolerance, high catalytic efficiency under alkaline conditions, strong substrate specificity (Patil and Kim 2018), and so forth due to their special habitat environment. The characteristics of marine microbial proteases are more in line with modern biotechnology and applications in different processing industries (Barzkar 2020; Bruins, Janssen, and Boom 2001; Homaei, Lavajoo, and Sariri 2016). For example, in the laundry detergent industry, cold‐adapted alkaline proteases are active at low temperatures and high pH values and can be used as detergent additives instead of chemical reagents or medium temperature proteases that are prone to denaturation under harsh conditions (Santiago et al. 2016). In biomedicine, cod trypsin has been shown to be 3–12 times more efficient in degrading large natural proteins than its thermophilic analogue bovine trypsin. The high digestibility of large natural proteins by cod trypsin adapted to cold conditions plays an important role in its efficacy against pathogens and positive effects on wounds (Gudmundsdottir, Hilmarsson, and Stefansson 2013). In the food industry, cold‐adapted enzymes from the ocean can be used for meat tenderization. Zhao et al. conducted an in‐depth study on the tenderization effect and mechanism of cold‐adapted collagenase MCP‐01 on beef at low temperatures (Zhao et al. 2012). These results indicate that the cold‐adapted collagenase MCP‐01 may have the potential to be used as a meat tenderizer at low and moderate temperatures. At present, the research focused on enzymatic method for allergen elimination mainly prefers to study the commercial proteases. However, elimination effect of existing commercial enzymes is relatively weak. Furthermore, only a few studies have investigated the application of marine proteases for the degradation of sensitized allergens (Barzkar et al. 2018; Yang et al. 2020). Therefore, isolating and utilizing protease genes from marine source are necessary for the degradation of sensitized allergic proteins in aquatic products.

In this study, we cloned and expressed the protease VSP2V‐280 from the *Virgibacillus* sp. SP2 strain, which was screened from traditional shrimp paste and can specifically degrade parvalbumin, and investigated its property of the protease and the degradation properties of parvalbumin. The findings of this study may serve as a reference for the degradation of parvalbumin in aquatic products and for improving the safety of aquatic products.

## 2 Materials and Methods

### 2.1 Materials

The protease producing strain *Virgibacillus* sp. SP2 was stored in our laboratory was used in this study. Receptive cell *Escherichia coli* DH5α, receptive cell *E. coli* BL21 (DE3), and pCold I vector were purchased from Tiangen Biochemical Technology Co. Ltd., China. Restriction enzymes Sac I and Xho I were procured from New England Biotechnology Corporation. Fish parvalbumin allergen enzyme‐linked immunoassay kit was bought from Qingdao Pribolab Biotechnology Company. *Ctenopharyngodon idella* , *Pelteobagrus fulvidraco* , *Parabramis pekinensis* , and *Carassius auratus* were bought from JIADEFU Supermarket, Lianyungang, China.

### 2.2 Methods

#### 2.2.1 Cloning of Marine Protease VSP2V‐280

DNA of *Virgibacillus* sp. SP2 was extracted by Bacterial Genomic DNA Extraction Kit (Tiangen). The protease gene was amplified through polymerase chain reaction (PCR) using the forward primer (5′‐CGAGCTCATGCAAGGGGCTATCGCCTTGAAAAA‐3′) and reverse primer (5′‐CCCTCGAGCACCTTGATACGGTTTAATCGTTTTT‐3′). Then, the target gene was enzymatically cut using restriction endonuclease (Sac I and Xho I) and linked to plasmid pCold I after cutting the plasmid by using the same endonuclease (Janatunaim and Fibriani 2020; Sugiki, Fujiwara, and Kojima 2017). The combined products were transformed into *E. coli* DH5α. Subsequently, transformed cells were screened using LB‐ampicillin medium. The positive transformants were selected, and then the plasmid was extracted and sent to Shanghai Sangon Biotech for verification (Ariyaei et al. 2019).

#### 2.2.2 Expression and Purification of Marine Protease VSP2V‐280 and Parvalbumin

The successfully sequenced plasmid was transformed into the *E. coli* BL21 (DE3) sensory state. The transformed positive clone was activated at 37°C and induced at 15°C for 24 h. The bacterial culture solution was centrifuged at 8000*g* for 15 min. After discarding the supernatant, the precipitated bacterial cells were resuspended in 0.1 M PBS buffer and washed twice by centrifugation at 8000*g* for 10 min. After the final wash, bacterial cells were resuspended in the appropriate volume of 0.1 M PBS buffer and subjected to ultrasonic crushing for 15 min (on for 3 s, off for 5 s; 20% W). After ultrasonic treatment, the solution was centrifuged at 10,000*g* centrifuge for 20 min and the ultrafiltrate (8 kDa). The supernatant was collected to obtain the crude enzyme solution.

The sequence of parvalbumin was obtained from a previous study (Sun et al. 2019). Pet28a was used as the carrier, with Ndel I and Xho I as the cleavage sites and the sequence was sent to Shanghai Sangon Biotechnology Company for synthesis. Synthesized plasmid was transformed into *E. coli* BL21 (DE3) receptive cells, and the transformed cells were screened using LB‐Kana medium. A single colony was selected and activated (OD: 0.6–0.8) at 37°C, and mixed with isopropyl‐β‐D‐thiogalactoside (50 mM). After induction at 37°C for 4 h, the bacterial solution was centrifuged at 4500*g* for 10 min, and the supernatant was discarded. Precipitated cells were washed with 0.1 M PBS buffer three times before adding the appropriate binding buffer (50 mM Tris, 300 mM NaCl, 0.2 mM phenylmethanesulfonyl fluoride [PMSF], and 0.1% Triton X‐100 [pH 8.0]). Cells was crushed by using ultrasonic cell crusher for 15 min (on for 3 s, off for 5 s; 20% W) and then centrifuged at 10,000*g* for 20 min to collect the supernatant.

Crude protease VSP2V‐280 and crude parvalbumin were purified by using Ni‐NTA affinity chromatography and SDS‐PAGE. Then, 5 mL of crude protein was injected into and passed through a nickel column at 4°C for 1 h to obtain the effluents. Imidazole eluent buffers of 20, 40, 60, 80, 100, 200, and 300 mM were used in sequence for elution. Protease VSP2V‐280 was subjected to SDS‐PAGE electrophoresis using 5% concentrated gel and 8% separator gel, while parvalbumin were subjected to SDS‐PAGE electrophoresis using 5% concentrated gel and 15% separator gel. The gel electrophoresis was run for 30 min at 80 V, followed by 120 V for 60 min (Sun et al. 2019). Subsequently, the gel was stained using dye of Coomassie brilliant blue. After staining for 30 min, decolorization was carried out by changing the decolorization solution every 30 min. After repeating the process for three times, The Bio Rad gel imager was used to observe and confirm whether the protein was purified.

#### 2.2.3 Determination of Protease Activity

Protease activity was determined according to Shaikh et al. with some minor modifications (Shaikh, Dixit, and Shaikh 2018). Then, 1 mL of the crude enzyme solution was incubated at 50°C for 10 min and then mixed with 1 mL of 1% (m/v) casein solution. The mixtures were incubated in a water bath at 50°C for 10 min. Subsequently, 2 mL of trichloroacetic acid (0.4 M) was added to the mixture, and the solution was further incubated for 10 min. After incubation, the solution was centrifuged at 7000*g* for 2 min and the supernatant was collected. Then, 1 mL of the supernatant was mixed with 5 mL of sodium carbonate (0.4 M) and 1 mL of folin‐phenol reagent. After putting the solution in a water bath at 50°C for 20 min for color development, its absorbance was measured at OD_680nm_ using an Eppendorf's full‐wavelength reader. The enzyme activity unit was defined as amount of enzyme required to produce 1 μg of tyrosine per minute under the specific conditions (Yang et al. 2020).

#### 2.2.4 Analysis of the Enzymatic Properties of Protease VSP2V‐280

##### 2.2.4.1 Effect of Temperature on Protease Activity

The optimum temperature for protease activity was determined between 20°C and 60°C, and the substrate was 1% (m/v) casein in 0.1 M PBS buffer (pH 8.0). For the thermostability, the protease was incubated between 20°C and 60°C for 1–5 h. The activities of protease were measured at optimal temperatures.

##### 2.2.4.2 Effect of pH on Protease Activity

The optimum pH of protease was determined by using 0.1 M sodium acetate buffer (pH 4–6), 0.1 M PBS buffer (pH 6–8), and 0.1 M Gly‐NaOH buffer (pH 8–11) (Yang et al. 2020). For pH stability, the enzyme was incubated in buffers of different pH values (pH 4–11) at the optimal temperature for 1 h, and activities of protease were measured.

##### 2.2.4.3 Effects of Metal Ions on Protease Activity

The effects of 1, 5, and 10 mM metal ions (Na^+^, K^+^, Ni^+^, Mg^2+^, NH_4_^+^, Zn^2+^, Ba^2+^ Co^+^, Ca^2+^, Mn^2+^, Cu^2+^, Fe^2+^) on the stability of protease were studied. Metal ions and proteases were incubated in a water bath at 50°C for 1 h. Measure residual activity under optimal conditions, and use protease activity without metal ions as a control.

##### 2.2.4.4 Effects of Inhibitors, Organic Solvents, and Surfactants on Protease Activity

To study the effects of inhibitors, surfactants, and organic solvents on enzyme activity, protease was combined with Ethylenediaminetetraacetic acid (EDTA), PMSF, DL‐dithiothreitol (DTT), SDS, and urea at the final concentration of 5 mM, and with glycerol, acetone, ethanol, methanol, dimethyl sulfoxide, ethyl acetate, acetonitrile, Tween80, TritonX‐100 at the final concentration of 1% (v/v). The solutions were incubated at 50°C for 1 h. Then, the protease activity of each solution was measured under optimal conditions, and the control was the protease without adding solvents.

#### 2.2.5 Parvalbumin Degradation Properties of Protease

##### 2.2.5.1 Effects of Enzyme Dose, Reaction Time, and Ca^2+^ on Parvalbumin Degradation

Incubate enzyme solutions of 18, 24, and 30 U/mL (without or with 1 mM Ca^2+^) with 0.3 mg/mL parvalbumin at 30°C and pH 8.0 for 0–7 h by diluting or ultrafiltration. Degradation of protein in the enzyme solutions was evaluated by SDS‐PAGE (15% separation gel, 5% concentrated gel).

##### 2.2.5.2 Sensitization Testing of Enzymatic Products

First, the standard curve was plotted based on the absorbance of cod protein standards (0, 4, 10, 40, and 100 μg/mL) measured at 450 nm using the Fish Parvalbumin Allergen Enzyme Immunoassay Kit. Based on the standard curve, the residual amount of parvalbumin was determined after incubation of parvalbumin with enzyme solution for 0–7 h.

#### 2.2.6 Sensitization Analysis of Enzymatic Hydrolysis Products

One gram of *C. idella* , *C. fulvidraco*, *P. pekinensis* and *C. auratus* were added into 5 mL of PBS buffer (20 mM, pH 7.5, containing 3% NaCl) and ground. The ground mixtures were then homogenized three times using a biological sample homogenizer at 8000 *g* for 30 s each time with 1 min interval. The homogenized solutions were centrifuged at 8000*g* for 10 min. After discarding the supernatants, the precipitates were collected and resuspended in 20 mM PBS buffer for four times to obtain fish homogenate. These homogenates were incubated with protease at a solid‐to‐liquid ratio of 1:45 and the residual amount of parvalbumin was measured at 0, 1, and 4 h using Fish Parvalbumin Allergen ELISA Kit. The fish with good enzymolysis effect was further studied to optimize the solid‐to‐liquid ratio. Enzyme solutions of 18, 36, 54, and 72 U/mL were incubated with 200 μL *C. idella* at ratios of feed to liquid 1:45. The residual amount of parvalbumin was measured at 0, 1 and 4 h using the kit.

#### 2.2.7 Protease Docking With Ca^2+^

Pubchem (https://pubchem.ncbi.nlm.nih.gov/) was used to find the two‐dimensional structure of Ca^2+^, and then Obgui V.2.3.2 software was used to determine its three‐dimensional structure. SWISS‐MODEL (https://swissmodel.expasy.org/) was used to model the three‐dimensional structure of VSP2V‐280. Pymol V.37 and Auto Dock Vina V.1.5.7 software were used for molecular docking. After removing the water molecules and treating the original ligands and receptors and molecules, active pockets were determined after hydrogenation. Subsequently, Vina was run to analyze and verify the minimum binding energy between the molecule and the target, and then Pymol V.37 was used to transform this information into a visual diagram.

#### 2.2.8 Data Processing

In this study, three parallel samples were tested during each experiment. SPASS V.26 was used for data analysis and Origin V.2018 was used to plot the graphs.

## 3 Results and Discussion

### 3.1 Cloning of the Marine Protease VSP2V‐280

**FIGURE 1:**
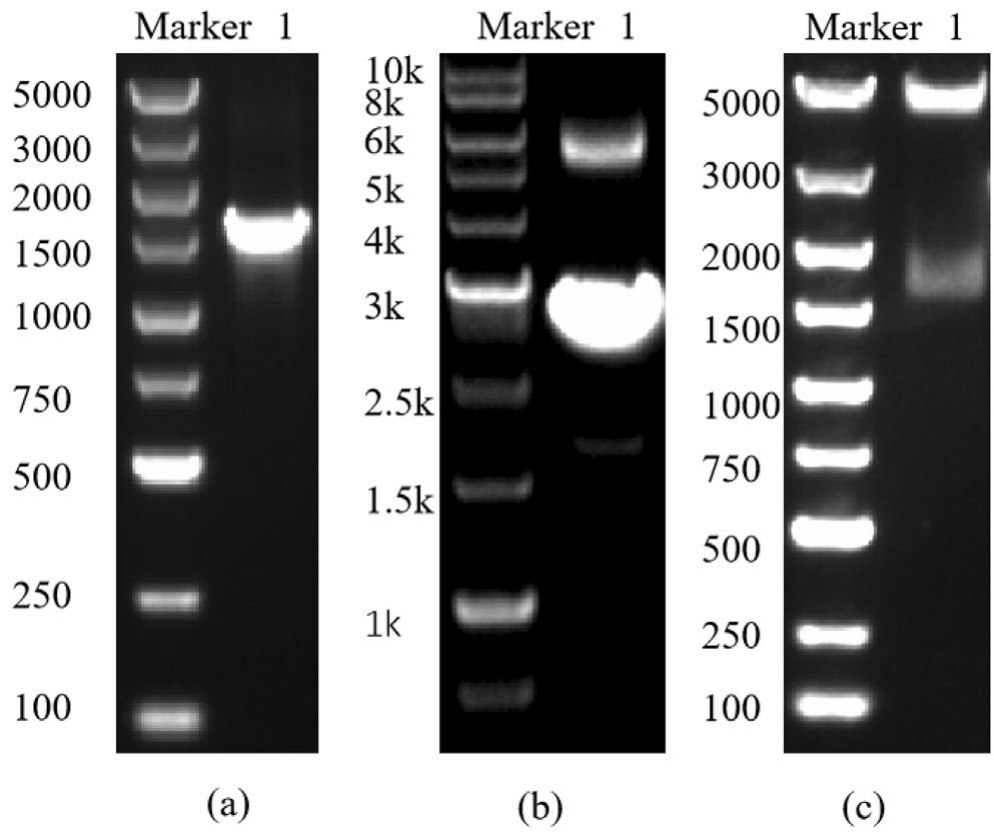
Cloning of marine protease VSP2V‐280: (a) amplified products of VSP2V‐280 target gene, (b) pCold I‐VSP2V‐280 clone plasmid, and (c) double digestion of pCold I‐VSP2V‐280 clone plasmid.

The gene of protease VSP2V‐280 was obtained by PCR using the genome of *Virgibacillus* sp. SP2 as a template. As shown in Figure 1a, the size of SP2‐280 gene was 1632 bp, which was consistent with the results of electrophoresis. Figure 1b shows the pCold I‐VSP2V‐280 clone plasmid, which has a size of 6015 bp. The recombinant plasmid was double‐digested by Xho I and Sac I to obtain two bands of 4407 and 1632 bp sizes, respectively, as shown in Figure 1c. Subsequently, the target gene was successfully inserted into pCold I vector.

### 3.2 Expression and Purification of Marine Protease VSP2V‐280 and Parvalbumin

**FIGURE 2:**
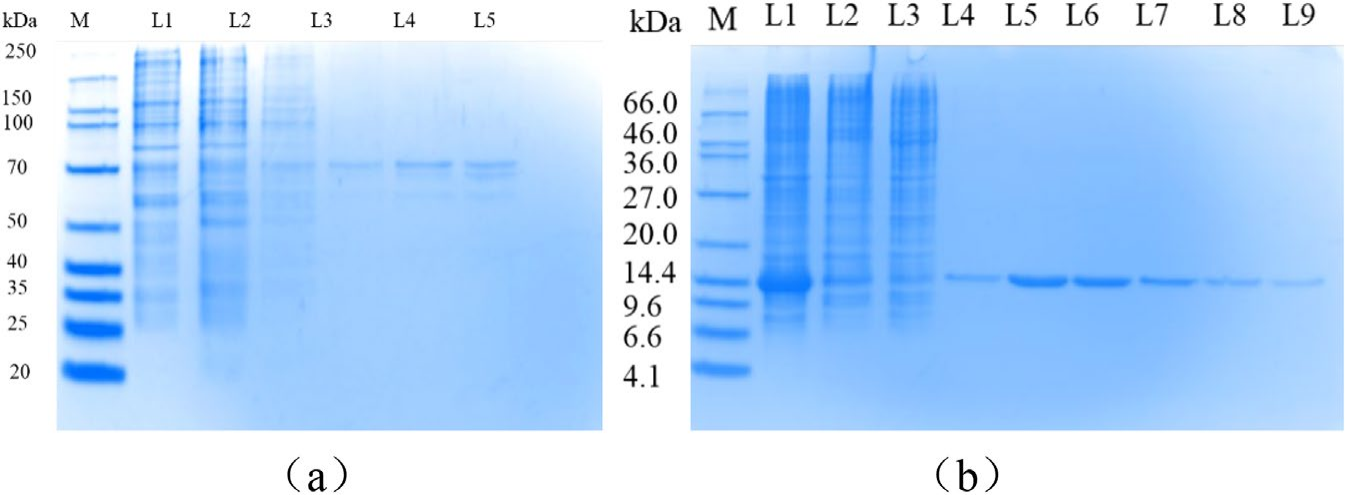
Purification of marine protease VSP2V‐280 and parvalbumin: (a) band diagram of marine protease VSP2V‐280 eluted using imidazole solutions of different concentrations; (b) band diagram of parvalbumin eluted using imidazole solutions of different concentrations. Here, L1: crude enzyme solution; L2: effluent; L3: 20 mM imidazole elution; L4: 40 mM imidazole elution; L5: 60 mM imidazole elution; and L6: 80 mM imidazole elution.

Total protein, enzyme activity, and the yield of purified protease VSP2V‐280 are shown in Table 1. The result showed that the final purification fold and yield of the protease were 1.54% and 36.8%, respectively. As shown in Figure 2a, the protein heterobands disappeared after purification by a nickel column and elution with imidazole solution of varying concentrations, leaving a single band near 58 kDa. Furthermore, protease VSP2V‐280 was successfully expressed and purified. Similarly, protein impurity bands disappeared after purification by nickel column and elution with imidazole of varying concentrations, leaving only a single band near 14.4 kDa (Figure 2b). This band indicated the pure parvalbumin, which has been reported to have a molecular weight of 10–14 kDa. Based on a previous study (Dumut et al. 2022) and the PAGE gel results, the purification of VSP2V‐280 and parvalbumin was verified.

**TABLE 1:** Purifications of protease VSP2V‐280.

Procedure	Total activity (U)	Total protein (mg)	Specific activity (U/mg)	Activity recovery (%)	Purification fold
Crude extract	421	150	2.8	100	1
Affinity chromatography	155	18.2	8.5	36.81	3.03

### 3.3 Biochemical Characteristics of Protease VSP2V‐280

#### 3.3.1 Optimum Temperature and Temperature Stability of Protease VSP2V‐280

**FIGURE 3:**
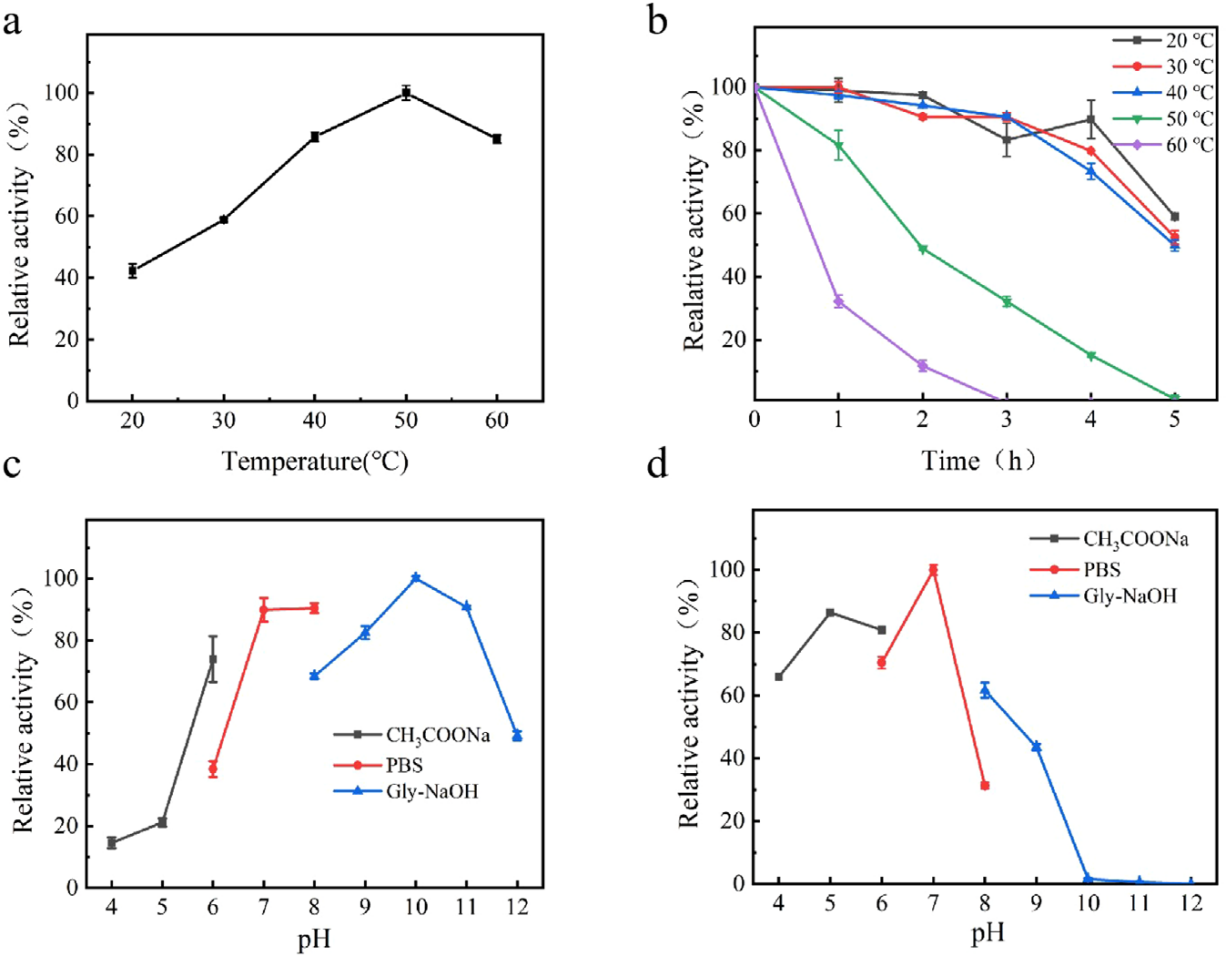
Effect of temperature (20°C–60°C) and pH (4–12) on marine protease VSP2V‐280: (a) effect of temperature on activity of VSP2V‐280, (b) temperature stability of VSP2V‐280, (c) effect of pH on activity of VSP2V‐280, and (d) pH stability of VSP2V‐280.

Analysis of the enzyme activity at different temperatures showed that VSP2V‐280 was active over a wide range of temperatures, with an optimal temperature of 50°C (Figure 3a). Remarkably, VSP2V‐280 maintained an activity of more than 40% at relatively low temperatures. In addition, the enzyme showed good thermal stability at medium and low temperatures (Figure 3b). After 4 h of incubation at 20°C–40°C, it still maintained more than 80% of the activity. However, when incubated at 60°C, the activity of protease decreased sharply with the increase in incubation time, which was similar to the trend shown by cold‐adapted serine proteinase in a previous study (Weijun et al. 2023). These results indicate the potential application value of protease in medium‐ and low‐temperature food processing processes.

#### 3.3.2 Optimum pH and pH Stability of Protease VSP2V‐280

The optimal pH and pH stability of VSP2V‐280 have been shown in Figure 3c,d. From the figure, it can be observed that the optimum pH of VSP2V‐280 is 10.0. The enzyme showed high enzyme activity in both neutral and alkaline environments, and was able to maintain more than 70% of activity at pH 6.0–11.0. In addition, VSP2V‐280 had the highest stability at pH 7.0 and good stability at pH 4–8, retaining more than 60% activity. This indicated the acid resistance of VSP2V‐280.

#### 3.3.3 Effects of Metal Ions on Protease Activity

**FIGURE 4:**
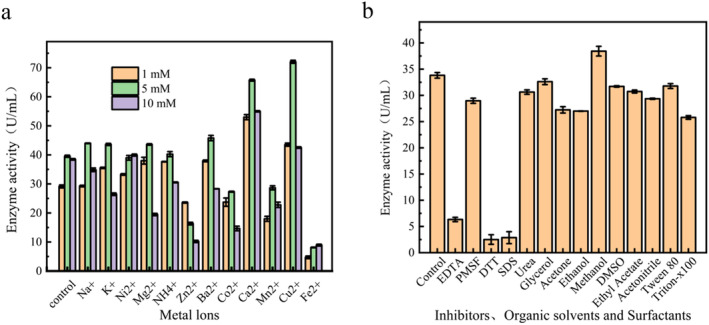
Effect of metal ions (1–5 mM), inhibitors (5 mM), organic solvents (10%), and surfactants (10%) on marine protease VSP2V‐280: (a) effect of metal ions on activity of VSP2V‐280 and (b) effects of inhibitors, organic solvents, and surfactants on protease activity.

Effect of metal ions on enzyme activity has been shown in Figure 4a. Ca^2+^ and Cu^2+^ significantly promoted the activity of protease. At Ca^2+^ concentrations of 1, 5, and 10 mM, protease activity increased by 182%, 166%, and 143%, respectively. On the other hand, 149%, 182%, and 110% increase in protease activity was observed after Cu^2+^ addition at 1, 5, and 10 mM concentrations, respectively. Furthermore, low concentration of Na^+^, k^+^, Ni^+^, Mg^2+^, NH_4_^+^, and Ba^2+^ also promoted the protease activity to some extent. However, Zn^2+^, Mn^2+^, and Fe^2+^ showed strong inhibitory effects on the enzyme.

#### 3.3.4 Effects of Inhibitors, Organic Solvents, and Surfactants on Protease Activity

Effects of inhibitors, surfactants, and organic solvents on protease activity have been shown in Figure 4b. EDTA significantly inhibited the activity of protease, indicating the importance of metal ions for enzyme activity. PMSF also inhibited the protease, which indicated that the purified protease was serine protease. Furthermore, DTT strongly inhibited VSP2V‐280, which indicated the presence of disulfide bonds in the protein structure. Organic solvents and surfactants had little effect on protease activity. Among these, methanol slightly promoted the activity of protease. After 1 h of incubation in organic solvents and surfactants, protease could retain over 80% activity.

### 3.4 Degradation of Parvalbumin by Protease VSP2V‐280

#### 3.4.1 Effect of Enzyme Dose, Reaction Time, and Ca^2+^ on Degradation of Parvalbumin

**FIGURE 5:**
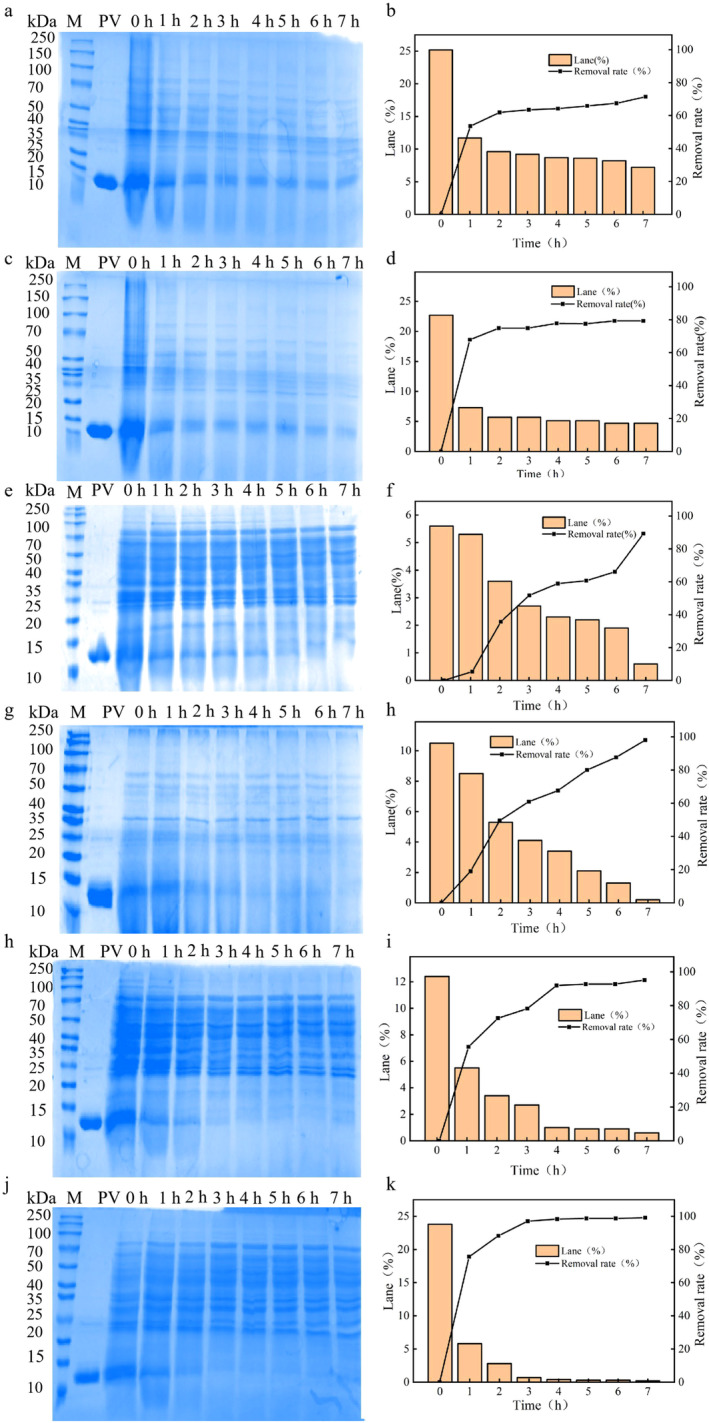
Parvalbumin degradation analysis by SDS‐PAGE gel assay under different concentrations of enzyme (18–30 U/mL) and presence/absence of Ca^2+^ (final concentration 1 mM): (a, b) 0.3 mg/mL of parvalbumin + enzyme activity of 18 U/mL without Ca^2+^; (c, d) 0.3 mg/mL of parvalbumin + enzyme activity of 18 U/mL with Ca^2+^; (e, f) 0.3 mg/mL of parvalbumin + enzyme activity of 24 U/mL enzyme without Ca^2+^; (g, h) 0.3 mg/mL of parvalbumin + enzyme activity of 24 U/mL with Ca^2+^; (i, j) 0.3 mg/mL of parvalbumin + enzyme activity of 30 U/mL without Ca^2+^; and (k, l) 0.3 mg/mL of parvalbumin + enzyme activity of 30 U/mL with Ca^2+^.

Figure 5 shows the effect of different doses of protease VSP2V‐280 and Ca^2+^ on the degradation of parvalbumin. The results showed that the parvalbumin band gradually weakened with the increasing concentration of enzyme. At an enzyme activity of 18 U/mL, parvalbumin still existed after 7 h, regardless the presence or absence of Ca^2+^. However, after adding Ca^2+^, parvalbumin band appeared shallower than the band without Ca^2+^ addition, and the degradation rate reached 80%. At an enzyme activity of 24 U/mL, parvalbumin was almost degraded at 7 h, with 90% degradation rate. At an enzyme activity of 30 U/mL, parvalbumin degradation rate reached 90% in just 4 h without Ca^2+^ addition and 97% in 3 h in the presence of Ca^2+^. These results suggested that the protease could effectively degrade parvalbumin, and Ca^2+^ effectively promoted the degradation process at a certain concentration. Similar to the reported alkaline proteases that were stable under alkaline conditions and could be significantly hydrolytic activated by Ca^2+^ (Hadjidj et al. 2018; Jeong, Baek, and Kim 2018). Tsai et al. showed that the immunoreactivity of parvalbumin and tropomyosin in *Nile tilapia* as well as orange‐spotted grouper were not significantly affected by boiling for 20 min, steaming for up to 10 min, baking at 200°C for 12 min, or deep‐frying at 160°C for 3 min (Jeong, Baek, and Kim 2018; Tsai et al. 2023). However, the immunoreactivity of parvalbumin was drastically reduced after 40 min of boiling and was completely lost after autoclaving. This suggests that autoclaving has some potential for reducing the allergy of fish parvalbumin. Then, there is no relevant literature showing the effect of protease on reducing the allergic parvalbumin. In the experiments, 0.3 mg/mL of parvalbumin was digested with 30 U/mL enzyme activity and the allergic rate of parvalbumin reduced 97% after 3 h, which suggested that the enzyme treatment also has a certain potential for hydrolysis of parvalbumin. In addition, marine bacterium *Virgibacillus* sp. SP2 contain eight protease genes in total, and we have cloned and expressed all of them. Only VSP2V‐280 could effectively degrade parvalbumin. The results suggest that corresponding proteases should be screened and investigated when degrading specific proteins.

#### 3.4.2 ELISA to Determine the Sensitization of Enzyme Digests

**FIGURE 6:**
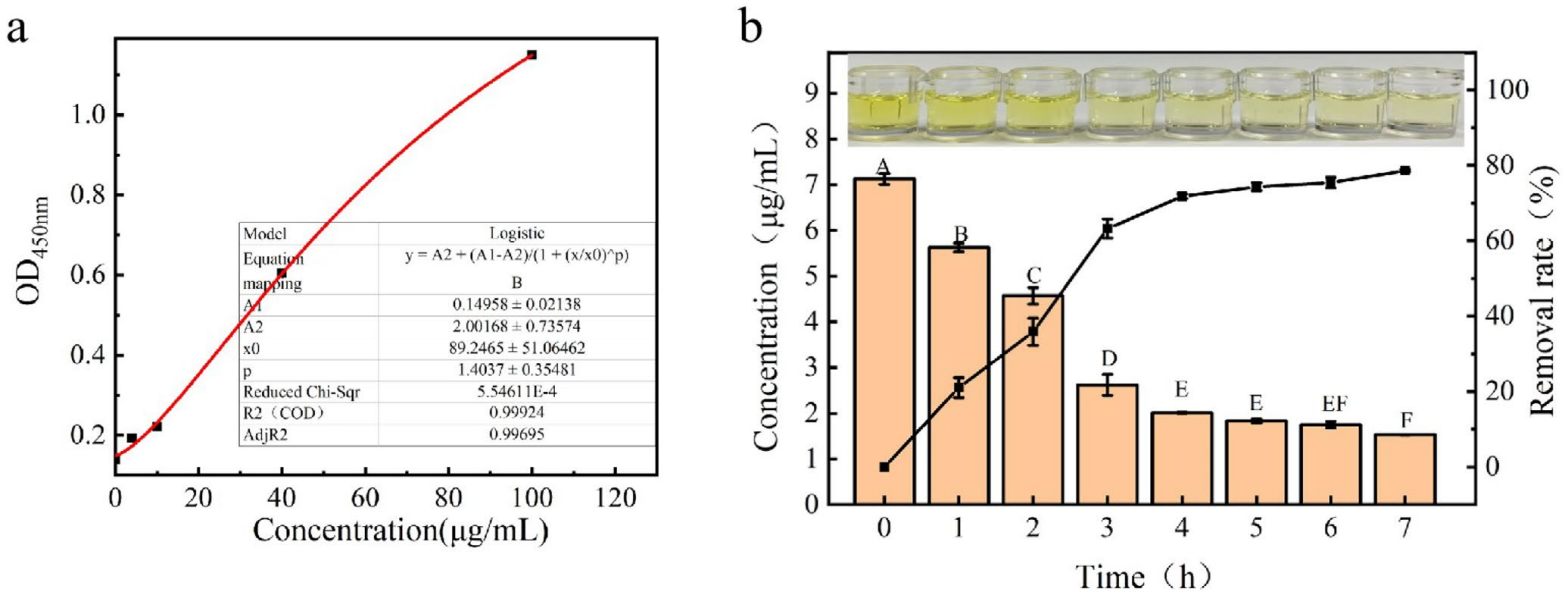
Standard ELISA curve for detection of parvalbumin and the residual amount of parvalbumin after digestion of 0.3 mg/mL of parvalbumin by protease (24 U/mL) under different conditions.

Standard ELISA kit was used to determine and draw the standard curve of parvalbumin. Four‐parameter method was used to fit the standard curve and the results have been shown in Figure 6a (Gadagkar and Call 2015; Nummer et al. 2018).

Figure 6b shows the results of ELISA, when 0.3 mg/mL of parvalbumin was hydrolyzed by 24 U/mL protease for 0–7 h. Around 63% parvalbumin removal was observed within 3 h, which further reached 78% at 7 h. These results were consistent with the findings observed through SDS‐PAGE. However, the sensitization of hydrolysate was still witnessed. The results showed that the basic protease VSP2V‐280 could effectively degrade the parvalbumin. Aquatic food is one of the most common foods worldwide to cause allergic reactions, which are potentially life‐threatening (Belmabrouk et al. 2023). The prevalence of food allergy varies considerably from country to country, but it appears to be predominant in areas where aquatic food is a staple food (Wai et al. 2021). In addition, as parvalbumin are the main source of fish allergy, it seems crucial to effectively remove it from fish products to reduce allergenicity. According to the results of ELISA, alkaline protease VSP2V‐280 could effectively degrade parvalbumin, providing a new approach for the removal of fish allergens.

### 3.5 Determination of Fish Sensitization by ELISA

**FIGURE 7:**
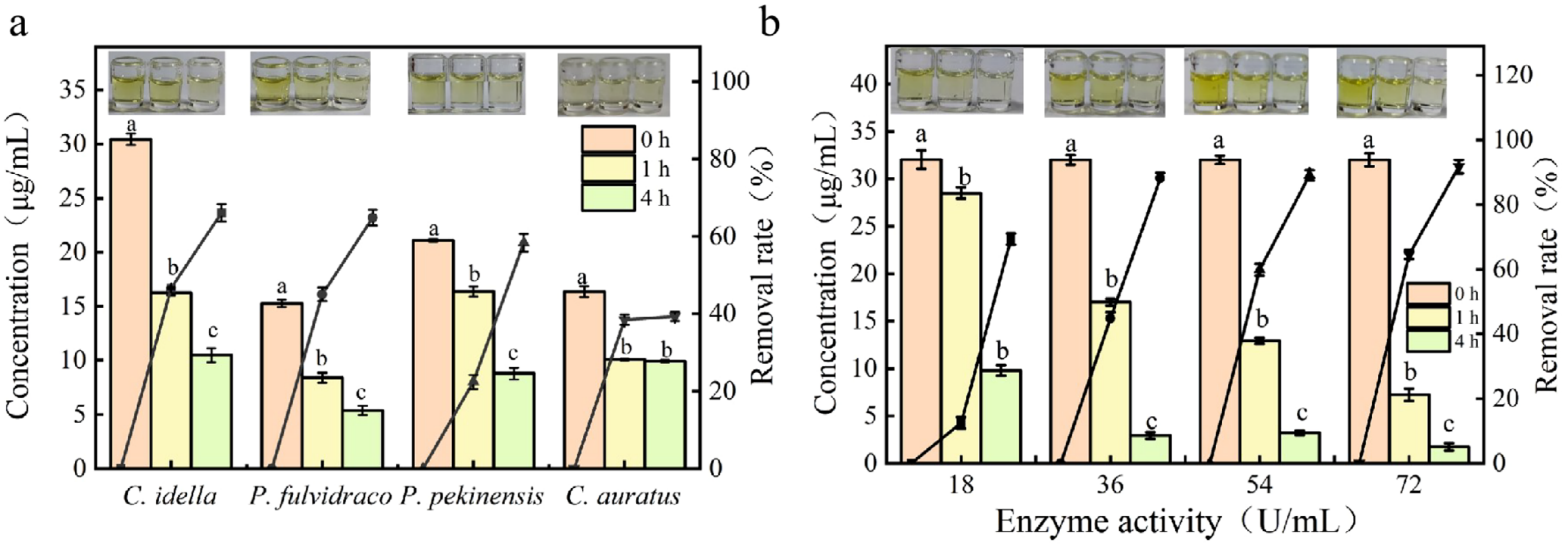
ELISA test of parvalbumin in fish: (a) parvalbumin content in different fish residues under 54 U/mL enzyme at 0, 1, and 4 h. (b) Residual parvalbumin in *C. idella* with different enzyme doses at 0, 1, and 4 h.

Figure 7a shows the residual amount of parvalbumin at 0, 1, and 4 h after hydrolysis of 200 μL of *C. idella* , *P. fulvidraco* , *P. pekinensis* , and *C. auratus* meat homogenates by enzyme activity of 54 U/mL VSP2V‐280. The figure shows that VSP2V‐280 showed the highest parvalbumin removal rates in *C. idella* and *P. fulvidraco* (46.65% and 45.04%, respectively, in 1 h and 66.09% and 64.86%, respectively, in 4 h). Parvalbumin removal rates in *P. pekinensis* and *C. auratus* were 58.40% and 39.31%, respectively, at 4 h. The low parvalbumin removal rate in *C. auratus* may be attributed to the relatively low parvalbumin content in *C. auratus* . Due to the positive effect of VSP2V‐280 on parvalbumin removal rate in *C. idella* , as well as considering the higher abundance of *C. idella* than other studied fish, the hydrolysis of *C. idella* by protease was optimized. Figure 7b shows that parvalbumin removal rate reached 11.8%, 46.11%, 61.18%, and 65.58%, respectively, in 1 h. when 200 μL of *C. idella* was hydrolyzed by proteases with enzyme activities of 18, 36, 54, and 72 U/mL, respectively. In 4 h, removal rates reached 69.53%, 89.33%, 88.68%, and 93.14%, respectively. This indicates the significant effect of protease on the sensitization of parvalbumin in fish.

### 3.6 Molecular Docking of Ca^2+^ With Protease VSP2V‐280

**FIGURE 8:**
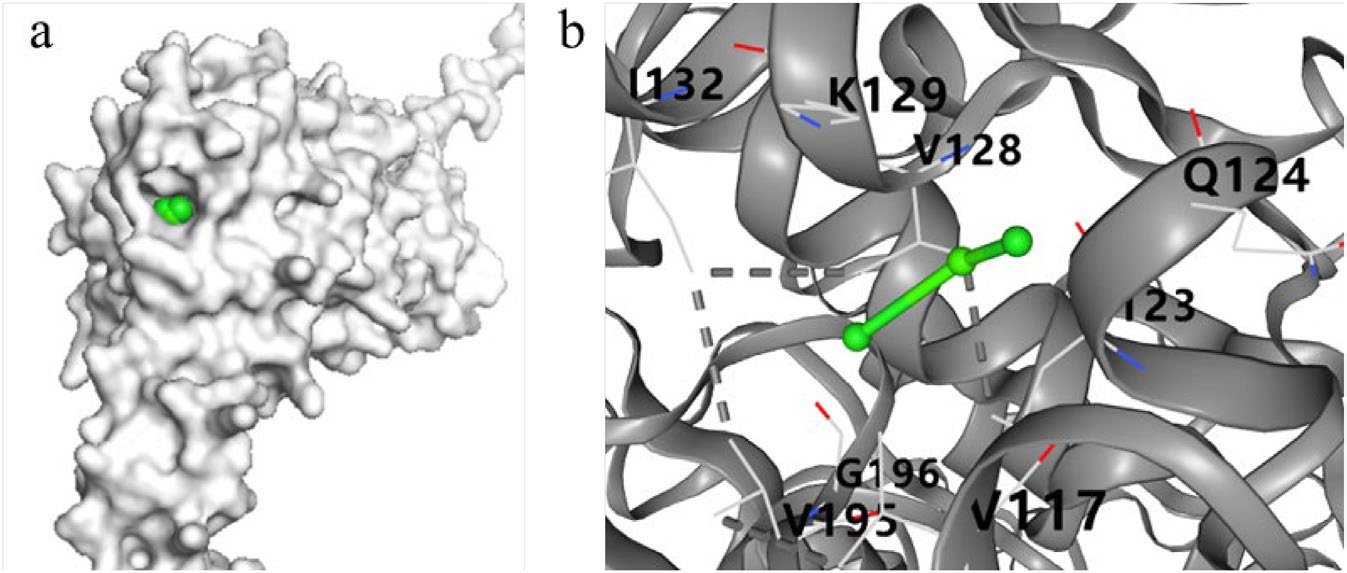
Molecular docking between Ca^2+^ and protease VSP2V‐280: (a) binding position of Ca^2+^ to VSP2V‐280 and (b) result of Ca^2+^ docking with VSP2V‐280.

When Ca^2+^ was docked with VSP2V‐280, five binding sites were observed. The conformation with lowest binding energy was selected as the optimal conformation. As shown in Figure 8, Ca^2+^ penetrated deep into the catalytic pocket of VSP2V‐280 and bound stably to the amino acids near the pocket. The binding energy of Ca^2+^ and VSP2V‐280 was observed to be −2.2, which was less than 0. This indicated that the binding of Ca^2+^ and protease was spontaneous, and there was a strong hydrophobic interaction between Ca^2+^ and protease. The interacting residues included I132, V195, V117, V128, and L123. It showed that the hydrophobic force was also important during the catalytic activity of protease VSP2V‐280. Zhang et al. studied the mechanism of interactions between Ca^2+^ and alkaline protease by determining the enzyme activity, particle size, and zeta point using fluorescence spectrometry and molecular docking (Zhang et al. 2019). The results showed that the binding of Ca^2+^ and alkaline protease not only promoted the enzyme activity, but also reduced the particle size of alkaline protease. Furthermore, binding of Ca^2+^ and alkaline protease promoted the stability of enzyme structure.

Metal ions can serve as cofactor for protease, thereby improving its activity (Song et al. 2023). Studies have shown that Ca^2+^ is the activator and stabilizer of most proteases. These ions bind to specific sites of protease to protect its conformation. Furthermore, Ca^2+^ ions significantly enhance the thermal stability of most alkaline proteases. Studies suggest that Ca^2+^ improves the activity of alkaline protease mainly by protecting the enzyme from thermal deformation at a certain temperature and avoiding conformational changes at high temperature (Michels and Clark 1997). Ca^2+^ addition leads to additional ion interactions, which promote the stabilization of enzyme. In this experiment, Ca^2+^ promoted protease activity to degrade parvalbumin. It was speculated that Ca^2+^ bound to the specific site of protease and protected its conformation, thus making it more stable and promoting the degradation of parvalbumin.

## 4 Conclusions

The protease VSP2V‐280 from *Virgibacillus* sp. SP2 could remove parvalbumin effectively. In this study, this gene was cloned and expressed in cold shock *E. coli* , and the resultant protease was used for parvalbumin degradation in fish. The optimum conditions for protease were temperature 50°C and pH 10.0. Ca^2+^ significantly promoted the activity of protease. Based on the gel analysis, when 0.3 mg/mL of parvalbumin was incubated with protease VSP2V‐280 (30 U/mL) containing 1 mM Ca^2+^ for 3 h, the parvalbumin removal rate reached 97%. In addition, 93.14% degradation of parvalbumin was observed on the enzyme activity of 72 U/mL protease incubated for 4 h with 200 μL of minced fish (1:45 ratio). The results reveal that protease VSP2V‐280 can efficiently remove the parvalbumin from fish meat and reduce its sensitization. This study suggests that protease VSP2V‐280 has great application prospects in the processing of fish products.

## Author Contributions

**Yue Bai:** investigation (equal), visualization (equal). **Yuan Gao:** data curation (equal), resources (equal). **Huili Tian:** data curation (equal), resources (equal). **Ming'ao Wang:** data curation (equal), resources (equal). **Xinxin Kang:** investigation (equal), visualization (equal). **Lei Zhang:** investigation (equal), visualization (equal). **Mingsheng Lv:** writing – review and editing (equal). **Shujun Wang:** funding acquisition (equal), project administration (equal).

## Conflicts of Interest

The authors declare no conflicts of interest.

## Data Availability

The datasets generated during and/or analyzed during the current study are available from the corresponding author on reasonable request.
